# Optimization of the feeding rate of *Anopheles farauti* s.s. colony mosquitoes in direct membrane feeding assays

**DOI:** 10.1186/s13071-021-04842-y

**Published:** 2021-07-07

**Authors:** Lincoln Timinao, Rebecca Vinit, Michelle Katusele, Louis Schofield, Thomas R. Burkot, Stephan Karl

**Affiliations:** 1 Papua New Guinea Institute of Medical Research, Madang, Papua New Guinea; 2 Australian Institute of Tropical Health and Medicine, James Cook University, Smithfield, QLD 4870 Australia

**Keywords:** Direct membrane feeding assay, *Anopheles farauti*, Papua New Guinea

## Abstract

**Background:**

Direct membrane feeding assays (DMFA) are an important tool to study parasite transmission to mosquitoes. Mosquito feeding rates in these artificial systems require optimization, as there are a number of factors that potentially influence the feeding rates and there are no standardized methods that apply to all anopheline species.

**Methods:**

A range of parameters prior to and during direct membrane feeding (DMF) were evaluated for their impact on *Anopheles farauti* sensu stricto feeding rates, including the starving conditions and duration of starving prior to feeding, membrane type, DMF exposure time, mosquito age, feeding in the light versus the dark, blood volume, mosquito density and temperature of water bath.

**Results:**

The average successful DMFA feeding rate for *An. farauti* s.s. colony mosquitoes increased from 50 to 85% when assay parameters were varied. Overnight starvation and Baudruche membrane yielded the highest feeding rates but rates were also affected by blood volume in the feeder and the mosquito density in the feeding cups. Availability of water during the pre-feed starvation period did not significantly impact feeding rates, nor did the exposure duration to blood in membrane feeders, the age of mosquitoes (3, 5 and 7 days post-emergence), feeding in the light versus the dark, or the temperature (34 °C, 38 °C, 42 °C and 46 °C) of the water bath.

**Conclusion:**

Optimal feeding conditions in *An. farauti* s.s. DMFA were to offer 50 female mosquitoes in a cup (with a total surface area of ~ 340 cm^2^ with 1 mosquito/6.8 cm^2^) that were starved overnight 350–500 µL of blood (collected in heparin-coated Vacutainer tubes) per feeder in feeders with a surface area ~ 5 cm^2^ (with a maximum capacity of 1.5 mL of blood) via a Baudruche membrane, for at least 10–20 min.

**Graphical Abstract:**

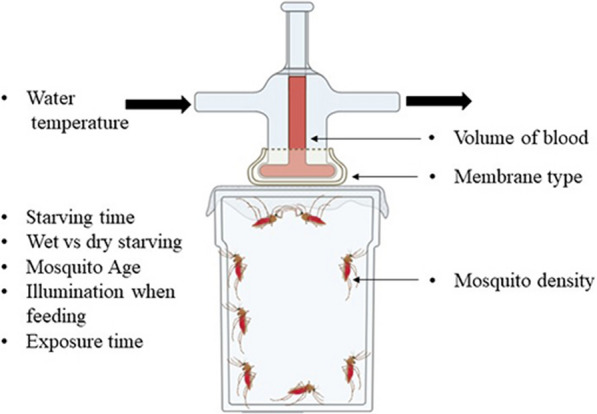

**Supplementary Information:**

The online version contains supplementary material available at 10.1186/s13071-021-04842-y.

## Background

In the malaria parasite life cycle, transmission through the mosquito vector represents a bottleneck where parasite populations shrink from millions in the human body to as few as one in the mosquito [1]. Thus, malaria transmission is vulnerable to interruption when transiting from the human to the mosquito host [2]. This transition can be studied by direct membrane feeding assays (DMFA). During DMFA, mosquitoes feed through a membrane on blood kept warm via water-jacketed glass feeders [3] including blood harvested from humans with circulating malaria parasites [4], to either study parasite development in the mosquito [4] or to test interventions that disrupt parasite development, hence interrupting transmission [5].

The mosquito blood feeding rate, i.e., the proportion of mosquitoes that successfully ingest blood, is an important determinant of overall infection success. The success rate of ingesting blood from a membrane feeder can vary depending on the mosquito species, whether the mosquitoes were collected in the wild [6] or reared in a colony [7], as well as the level of adaptation of the colony.

**Fig. 1:**
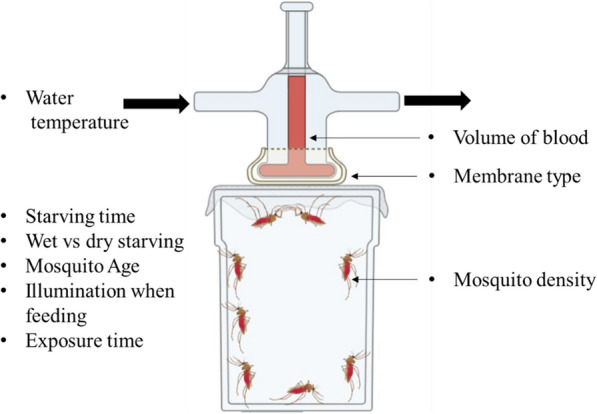
Membrane feeding assay set-up with the parameters impacting the feeding success of mosquitoes on the direct membrane feeders (figure created using BioRender.com)

Blood feeding rates also depend on the experimental conditions under which the DMFAs are conducted, including (i) the duration of starvation before exposure, (ii) the starving conditions (access to water or no access to water), (iii) the type of membrane used, (iv) the amount of time mosquitoes are allowed to feed, (v) the mosquito age, (vi) feeding in the light or in the dark, (vii) the blood volume in the feeder, (viii) the density of mosquitoes attempting to feed and (ix) water bath temperature during DMFA (Fig. 1). Other parameters which also potentially influence blood-feeding rates in DMFA but were not investigated here include the blood meal source [8], the haematocrit level, [9] and phagostimulants such as sodium chloride and sodium bicarbonate for *Anopheles* species [10].

However, membrane feeding studies have been conducted with a range of conditions and with varied feeding success [11, 12]. Thus, there is a need to optimize DMFA conditions for each colony mosquito species.

Starving conditions are a key component that greatly impact mosquito feeding rates and a balance needs to be established between starving the mosquitoes for too long, thereby increasing mosquito mortality or affecting their fitness [13], and not starving for long enough so mosquitoes only partly feed or not at all.

Most studies describe dry starving for durations from 5 to 36 h [12, 14–16] while other studies performed starving where the mosquitoes had access to water for 12 h [17, 18]. A study conducted by Coulibaly and colleagues compared the feeding rates of mosquitoes dry starved 8 h, 14 h and 20 h and concluded that mosquitoes starved 8–14 h yielded significantly higher feeding rates than mosquitoes starved 20 h [13]. However, most studies did not directly report the impact of starving on the feeding rate.

Membranes take the role of an artificial skin in the feeding experiments. An ideal membrane will yield the highest feeding rates in the shortest period of time. Parafilm and natural membranes such as Baudruche, sausage casing, chicken skin, or rat skin have been used [6, 19]. Natural membranes which closely mimic the skin resulted in the highest feeding rates followed by Baudruche membrane, which is derived from bovine cecum, and finally Parafilm, a wax synthetic membrane [3]. Most studies reported using Baudruche membrane [11, 20, 21] while others used Parafilm membrane [16, 22]. Interestingly, a study done by Coulibaly and colleagues showed that there was no significant difference between the feeding rates, survival and infection rates from feeding experiments with either Baudruche or Parafilm membranes, for *Anopheles coluzzii* mosquitoes [13].

Mosquitoes 2–8 days post-emergence have been used in different studies [6, 7, 12, 13, 16, 17, 23–25]. The main consideration in this is that mosquitoes are fed at an early age so that they survive for the required duration for either oocysts [11, 12, 16, 26, 27] or sporozoites [11, 26, 27] to develop. The study by Coulibaly and colleagues is so far the only one that compared the feeding rate of *An. coluzzii* mosquitoes between 3 and 9 days post emergence. The authors determined that 3-day-old mosquitoes had a significantly higher feeding rate compared to 6 and 9-day-old mosquitoes [13].

Mosquito density is another factor that may influence the mosquito feeding rate. Rutledge and colleagues observed that having more mosquitoes per cage can result in lower feeding rates [3] and crowding, making handling, especially removing of unfed mosquitoes, difficult. Vallejo and colleagues observed that 100 *An. coluzzii* mosquitoes per cage (or 1 mosquito per 5 cm^2^) resulted in the highest *Plasmodium vivax* infection prevalence after DMFA [27]. However, the study did not report on the feeding rate of the different mosquito densities in relation to infection success.

Little has also been reported with respect to the impact of the other parameters listed above on the feeding rates. Much of the focus is on the infection rates. Therefore, the focus of this study was to determine the optimal feeding conditions for *Anopheles farauti *sensu stricto colony mosquitoes in order to maximize their feeding rates in DMFA.

## Methods

### Mosquito colony maintenance

The *An. farauti* s.s. mosquito colony was derived from a colony established in 1968 in Rabaul, East New Britain province. [28] The laboratory colony was maintained at 28 ± 8 °C and 68 ± 25% relative humidity. The light cycle is approximately 11 h dark and 12 h light, including a 30 min dusk and 30 min dawn period. The larvae were fed ground fish food (Marine Master Tropical Fish Flakes, Australia) while the adults were provided 10% sucrose (Ramu Sugar, Papua New Guinea) solution available as soaked cotton wool balls placed on top of the mosquito cages. To maintain the colony, uninfected blood (no malaria) was obtained from donors following informed consent procedure. Occasionally, direct skin feeding was used to maintain the colony.

### Direct membrane feeding assays

**Fig. 2:**
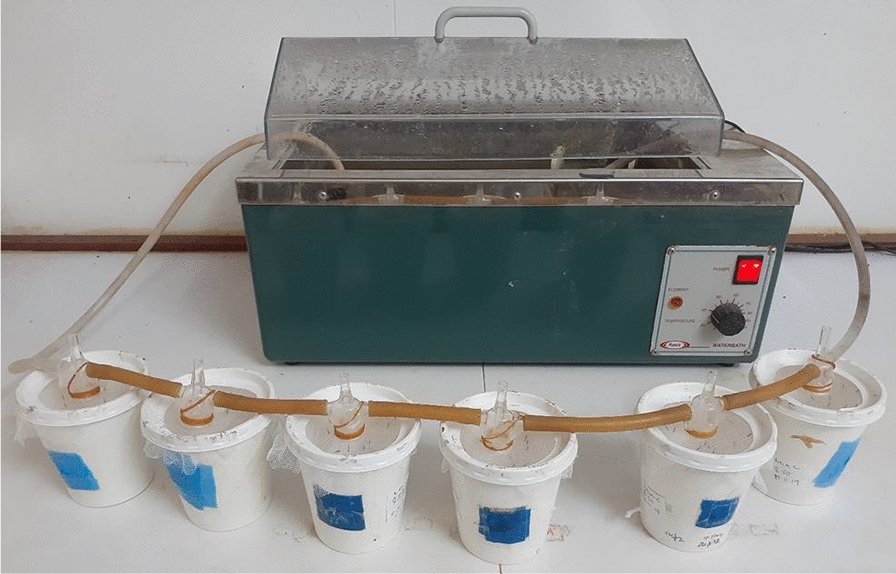
Direct membrane feeding assay (DMFA) set-up. Cups connected in a series by tubes to a mini aquarium pump within the water bath which is maintained at ~ 38 °C

Water-jacketed glass membrane feeders were connected in series by rubber hoses to a mini aquarium pump placed inside a 37–38 ºC water bath (Fig. 2). Unless otherwise stated, all trials used an average of 5-day-old mosquitoes with 50 female *An. farauti* s.s. placed in a cup (surface area of ~ 340 cm^2^ with a total volume of ~ 476 cm^3^) and offered blood meals from feeders with a diameter of 2.5 cm (a surface area of ~ 5 cm^2^ with a maximum blood volume capacity of 1 mL). The duration of overnight starving ranged between 18 and 21 h.

The experiments were done sequentially with a single parameter being varied and tested, incorporating the optimal conditions of the preceding tests. Following the feeding experiments, unfed mosquitoes were separated from the fully fed mosquitoes and the feeding rate calculated. Data were transformed using arcsine (p) [29] prior to performing paired *t* tests to test the significance of the difference observed between groups. Analysis of variance (ANOVA), followed by *t* tests, was used to test for significant variation between more than two groups. A flow chart summarizing the parameters tested is provided in Additional file 1: Table S1.

### Starving time

*An. farauti* s.s. were dry starved (no access to sugar or water) for 2 h, 4 h, 6 h and overnight (~ 21 h) and compared to a control of mosquitoes exposed without starving. Mosquitoes were exposed to ~ 750 µL of blood for 30 min in the dark (with a black piece of blanket draped over the membrane feeder) following starvation. Parafilm membrane (cut into 3 cm × 3 cm and stretched to ~ 5 cm × ~ 5 cm) was used to feed the mosquitoes. The Parafilm was not exposed to human odour prior to feeding. Ten replicates were performed.

### Type of starving: access to water versus dry (no access to water)

*An. farauti* s.s. mosquitoes were starved overnight, with one cup of mosquitoes having access to cotton soaked in water while the other did not have access to water (dry starved). The mosquitoes were then given access to ~ 750 µL of blood using a Parafilm membrane for 30 min in the dark. Five replicates were performed.

### Membrane type

Two membrane types were tested, namely Parafilm and Baudruche membranes. The Parafilm membrane was standardized by cutting it into 3 cm × 3 cm pieces and stretching to ~ 5 cm × ~ 5 cm. Mosquitoes were dry starved overnight. The mosquitoes were given access to ~ 750 µL of blood in a membrane feeder for 30 min in the dark. Eight replicate experiments were performed.

### Exposure time

Exposure times of 10 min, 20 min and 30 min were evaluated following dry overnight starving and the mosquitoes were then exposed to ~ 750 µL of blood for the specified time. Baudruche membrane was used to feed the mosquitoes. Eight replicate experiments were performed.

### Mosquito age

Mosquitoes aged 3, 5 and 7 days were tested in nine replicates. The mosquitoes were starved overnight before exposure to ~ 750 µL of blood via a Baudruche membrane for 20 min in the dark﻿.

### Feeding in the light or in the dark

A total of 2 cups of mosquitoes were prepared and starved overnight. One cup of mosquitoes was fed with the net top exposed to ambient room lighting, while the second cup had a black blanket placed over it while they were exposed to ~ 750 µL of blood via Baudruche membrane for 20 min. Seven replicate experiments were performed.

### Volume of blood

Blood volumes of 125 µL, 250 µL and 500 µL in a water-jacketed glass feeder of 1.5 mL maximum capacity were tested to determine the minimum blood volume which could yield high feeding rates. Three cups of 6-day-old (mean age) *An. farauti* s.s. were prepared, starved overnight and allowed 20 min to feed in the dark at each blood volume via a Baudruche membrane. Six replicate experiments were performed.

### Mosquito density

Three different mosquito numbers, i.e., 20, 50 and 100 per cup, were tested. Mosquitoes at a mean age of 4 days were dry starved overnight. The mosquitoes were allowed to feed on ~ 500 µL of blood for 20 min under illuminated conditions via a Baudruche membrane. Six replicates were performed for this test.

### Water temperature

Four different water bath temperatures were tested, i.e., 34 °C, 38 °C, 42 °C and 46 °C. Mosquitoes were dry starved overnight before being allowed to feed on ~ 500 µL of blood for 20 min under illuminated conditions via a Baudruche membrane. Seven replicates were performed for this test.

## Results

The volunteers who donated blood for membrane feeding were adults with a median age of 46 (range of 28–52) and a median haemoglobin level of 15.5 g/dL with the range of 11.1–17.7 g/dL. The median room temperature was 28.05 °C (range of 22.2–30.34 °C) with a relative humidity of 78.38% (range of 56–89.3%). The baseline parameters to which subsequent tests were compared were: 50 female *An. farauti* s.s. per cup, aged between 3 and 5 days, dry starved between 0 and 4 h, and fed in the dark using Parafilm as the membrane for ~ 30 min, with 750 µL of blood at a water bath temperature of ~ 38 °C.

Table 1 summarizes the results of the analysis for the starving duration, type of starving, membrane type, feeding duration, mosquito age, feeding in the light versus in the dark, volume of blood, mosquito density and water bath temperature.

**Table 1:** Mosquito feeding rate according the feeding parameters being tested

Feeding parameters	Total number of mosquitoes in cups	Total number fed	Total unfed	Empirical average feeding rate (%)*	Range (%)	*p* value**
Starving time						
0 h	916	240	676	27	4–45	< 0.01
2 h	864	288	576	34	15–56	< 0.01
4 h	933	368	565	40	20–54	< 0.05
6 h	954	405	549	45	23–82	< 0.01
Overnight (~ 21 h)	841	492	349	60	19–97	Ref
Type of starving						
Access to water	217	134	83	62	43–89	0.47
Dry starving	234	167	67	71	31–92	Ref
Membrane type						
Baudruche	476	389	87	85	70–100	< 0.05
Parafilm	457	268	189	53	42–76	Ref
Exposure time						
10 min	426	326	100	77	63–98	0.63
20 min	469	368	101	80	43–100	0.61
30 min	386	314	72	81	72–89	Ref
Mosquito age						
3 days	631	447	184	75	39–96	0.38
5 days	606	473	133	81	50–93	Ref
7 days	613	449	164	75	55–90	0.08
Light/Dark						
Light	317	274	43	85	64–96	0.88
Dark	326	276	50	84	63–96	Ref
Volume of blood						
125 µL	289	190	99	65	50–88	< 0.05
250 µL	272	229	43	84	67–96	0.54
500 µL	295	256	39	87	77–98	Ref
Mosquito density						
20 mosquitoes	194	148	46	76	64–79	0.13
50 mosquitoes	480	410	70	85	79–87	Ref
100 mosquitoes	927	696	231	75	67–74	< 0.01
Water bath temperature						
34 °C	330	271	59	82	51–95	0.23
38 °C	335	299	36	89	81–100	Ref
42 °C	320	277	43	86	72–98	0.61
46 °C	305	244	61	79	58–96	0.09

Ref: reference group for the calculation of the *p* values

*Empirical averages were calculated as the average feeding rate of all replicates obtained for a specific condition

**Significantly different from the reference when *p* < 0.05. Data were transformed using arcsine (p) prior to doing the paired *t* test

**Fig. 3:**
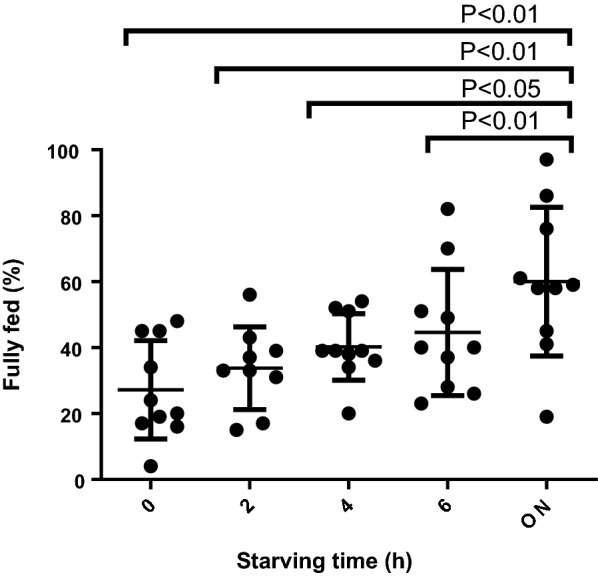
Proportion of mosquitoes that fed following the different starving times. The differences between the starving times of 0–4 h and overnight (ON) starving were statistically significant. The error bars denote means and standard deviations. *p* values denote an arcsine transformation of the data

There was a statistically significant difference observed between the feeding rates of the different starving times as determined by one-way ANOVA (*F*(3, 24) = 8.982, *p* < 0.001). When performing *t* tests for the different paired groups, we found that there were significant differences between the overnight starving and the other starving times (Fig. 3), with feeding rates approximately doubled when comparing from 0 h (27%) to ~ 21 h (60%). The differences in the feeding rates after starving the mosquitoes for 6 h, 4 h and 2 h were not statistically significant (*p* = 0.51, *p* = 0.10, *p* = 0.18). We observed a mortality rate of 7% with the overnight starvation compared to the other groups, which exhibited an average mortality of 1%. The observed difference between the mortality rates at starving times of 0 h, 2 h, 4 h, 6 h and overnight is statistically significant as determined by the one-way ANOVA (*F*(3, 19) = 4.000, *p* = 0.03). There was no significant difference observed between the type of starving, i.e., whether the mosquitoes were dry starved or allowed to feed on water during the starving period (*p* = 0.47).

**Fig. 4:**
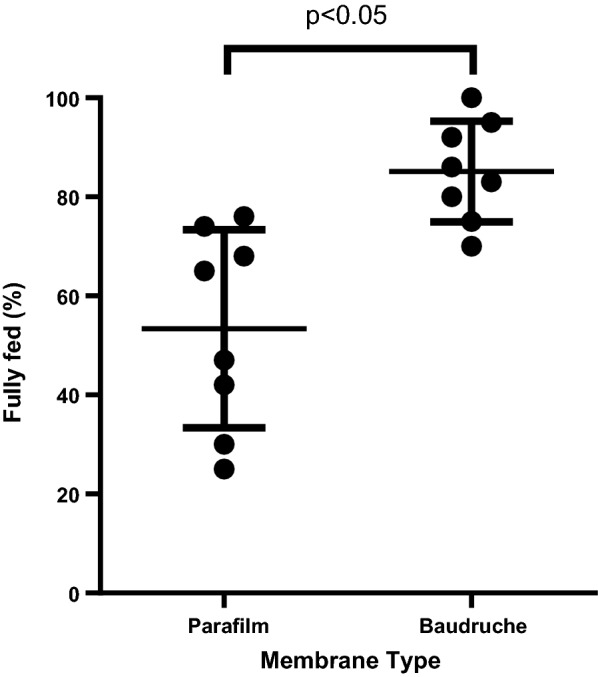
Proportion of mosquitoes that were fully fed when using Parafilm and Baudruche membrane types. The observed difference in the performance of Parafilm and Baudruche membranes is statistically significant (*p* < 0.05). The error bars represent means and standard deviations

There was a statistically significant difference in the feeding rates for the two types of membranes tested, Baudruche and Parafilm (*p* < 0.05) (Fig. 4). Feeding rate increased to 85% when the Baudruche membrane was used.

**Fig. 5:**
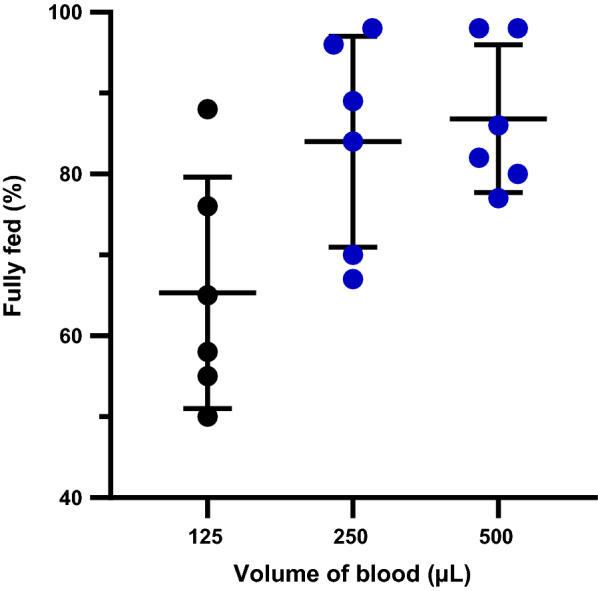
Feeding rates for varying blood volume. A significantly higher proportion of mosquitoes fed on 250 µL and 500 µL of blood compared to the feeding rate for 125 µL of blood (p < 0.05). The error bars are means with standard deviations. The groups represented by blue dots were significantly different from the group represented by black dots

**Fig. 6:**
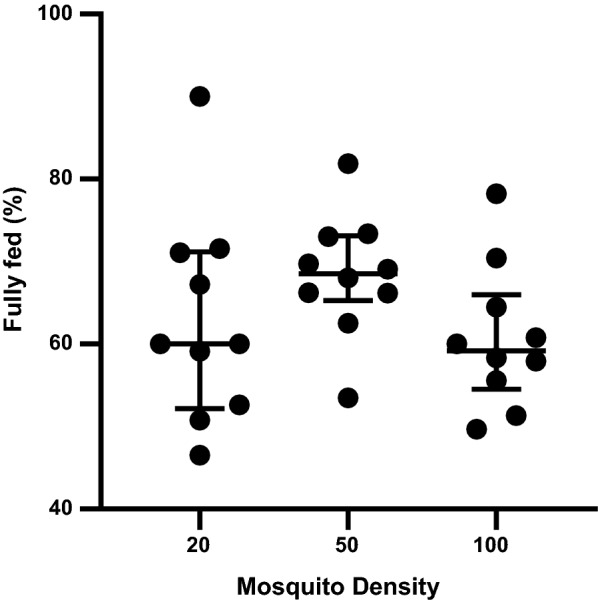
Feeding rates for different mosquito densities. The feeding rates for 50 mosquitoes per cup were significantly higher than for 100 mosquitoes per cup (*p* < 0.01). The error bars represent means and standard deviations

Exposure times, mosquito age or feeding in the light versus in the dark were not observed to significantly influence membrane feeding rates (Table 1). However, there was a statistically significant difference in the feeding rates for the different volumes of blood as determined by one-way ANOVA (*F*(2, 10) = 13.70, *p* < 0.01). We observed an increase in the feeding rate when the blood volume was increased from 125 µL to anything above 250 µL. The difference was statistically significant (*p* < 0.01) (Fig. 5). The differences in the feeding rates at different mosquito densities approached significance as determined by one-way ANOVA (*F*(2, 15) = 3.861, *p* = 0.052). When comparing different mosquito density groups, we observed that feeding rates were higher for cups with a mosquito density of 50 per cup as compared to 100 per cup (*p* < 0.01) (Fig. 6). However, there was no significant difference between 50 and 20 mosquitoes per cup (*p* = 0.13), indicating that 50 mosquitoes per cup was closer to the optimal mosquito density. Interestingly, we did not observe any statistically significant difference in the feeding rate between 20 and 100 mosquitoes.

There was no statistically significant difference between the feeding rates at different exposure times (10, 20 and 30 min), the different mosquito ages (3, 5 and 7 days old) and the different water bath temperatures (34, 38, 42 and 46 °C) as determined by one-way ANOVA (*F*(1, 9) = 0.3522, *p* = 0.63, *F*(2, 13) = 1.197, *p* = 0.32, *F*(1, 9) = 0.3522, *p* = 0.63), respectively.

## Discussion

In this study, a selection of parameters that could potentially influence the feeding rate of *An. farauti* s.s. colony mosquitoes in DMFAs were investigated to identify the optimal conditions to enable high feeding rates. By systematically varying individual parameters sequentially, the baseline feeding rate of ~ 50% was increased to ~ 85%.

Two parameters in particular were associated with improved feeding rates: the starving duration prior to membrane feeding and the membrane type used during the feed. Increasing the starving duration to overnight (~ 21 h) resulted in a statistically significant difference in the feeding rate (60%) in comparison to the other starving durations (27–45%). We selected overnight starving, as there were no significant differences between the 6 h and 4 h or 2 h (*p* = 0.51, *p* = 0.10), which had lower feeding rates. However, we did observe a significantly higher mortality rate with overnight starving (7%) as compared to the other starving times (1%). This may be due to the long hours of starving. However, the high feeding rate compensates for the increased mortality rate. When compared to the average feeding rate of the other starving times (2 h, 4 h and 6 h) of 39%, overnight starving resulted in a higher feeding rate by a factor of 1.5. Overnight starving was used in several previous studies [13, 16, 22, 27, 30], while some studies used a minimum of 5 h of starvation with various *Anopheles* mosquito species [12, 20]. The use of a Baudruche membrane together with overnight starving further increased the feeding rate to 85%. Previous studies have used either Baudruche membrane or Parafilm for performing DMF [11, 16, 20–22] with different mosquito species. A study by Coulibaly and colleagues comparing the two membrane types using *An. coluzzii* mosquitoes showed that there was no significant difference between the Baudruche and Parafilm membranes when adjusting for other covariates [13]. The superior performance of Baudruche membrane over Parafilm observed here may be due in particular to the fact that the Baudruche membrane is made from a natural material, as previous evidence has shown that natural membranes have better feeding performance [31]. Another possibility is that the natural membrane was favoured because it more closely resembled the direct skin feeding, which is used to feed the mosquitoes for colony maintenance. Varying the conditions of the other feeding parameters (e.g., feeding in the light versus in the dark, mosquito age, blood volume, feeding duration and water bath temperature) did not significantly increase the feeding rate further.

Even though there was no significant increase in the feeding rates when testing the other parameters, our chosen selection contributed towards the economical use of resources. It was observed that feeding mosquitoes for 10 min yielded similar feeding rates as feeding for 20–30 min. This is within the range of feeding times between 10 and 30 min that have been used in different studies, and represents a significant time saving [11, 21, 30, 32]. We chose to use 10–20 min depending on our feeding schedule. The optimal volume of blood used per glass feeder is important, especially when working with limited amounts of infected blood. A volume of 350 µL was recommended for the size (2.5 cm in diameter, surface area of ~ 5 cm^2^) of the glass feeders used in the present study [11], while another study used a total of 1.5 mL [20] using the same size feeders. However, volumes between 250 and 500 µL yielded similar high feeding rates. As such, we chose to use any volume within the range of 250–500 µL, depending on the total volume of blood available to feed the mosquitoes. This represents a threefold decrease in the volume of blood used compared to the original volume of 750 µL.

With respect to mosquito density, it was observed that 50 mosquitoes (approximate density per cup of 1 mosquito/6.8 cm^2^) feeding on a ~ 5 cm^2^ membrane surface area yielded a high feeding rate, while 100 mosquitoes per cup (i.e., 1 mosquito/3.4 cm^2^) resulted in lower feeding rates. The difference was statistically significant (*p* < 0.01). There was no significant difference in the feeding rates when comparing densities of 20 mosquitoes and 50 mosquitoes per cup. Interestingly there was also no significant difference between 20 and 100 mosquitoes per cup. This observation could imply that there is really no significant difference between the three mosquito densities, as indicated by the ANOVA test (*F*(2, 15) = 3.861, *p* = 0.052). However, the *p* value observed here is approaching statistical significance, indicating that more tests need to be conducted in order to conclude whether the difference observed between the mosquito densities is significant. Established protocols recommend using 50–100 mosquitoes for similar-sized cups depending on the size of the feeder and the feeding rate [11, 12]. Based on these results, we chose to use 50 mosquitoes per cup to maximize the number of mosquitoes exposed to blood and reduce the risk of a crowding effect during feeding. Although various groups have preferred using one or the other of the two types of starving conditions, either dry or by exposing the mosquitoes to water prior to feeding [12, 14–18], we did not observe any significant difference between the two. It may be that *An. farauti* s.s. mosquitoes do not feed on water as efficiently as they would on sugar solution, as the mosquitoes have specialized sensory organs that detect the presence of blood or nectar, triggering them to feed [33]. Hence, we observed no significant difference between the feeding rates of the starving conditions. We opted for dry starving.

We also did not observe any significant difference between feeding the mosquitoes when exposed to light or in the dark. However, other studies indicated that their membrane feeding experiments were performed in the dark [20, 22]. Also, a protocol by Ouedraogo and colleagues indicated that DMF should be performed in the dark to mimic the natural feeding conditions during the night, while membrane feeding experiments are commonly undertaken during the day [12]. Our contrasting observation may be because this mosquito species has been colonized for over 50 years and feeds well regardless of the light conditions. Usually in the wild the *An. farauti* s.s. mosquito would feed in the evening starting at 6 pm and peak between 10 and 11 pm when it is dark [34]. Here we chose to feed the mosquitoes while exposing them to the light, as it is easier to monitor the progress of the mosquitoes feeding.

Furthermore, we did not detect any significant differences in the feeding rates between age groups 3-, 5- and 7-day-old mosquitoes. In contrast, Coulibaly and colleagues noted a significant difference in the feeding rates between 3 days and 6–9-day-old *An. coluzzii*. It could be that this age-dependent behaviour change is species- or colony-specific. Collectively, studies on various *Anopheles* mosquito species reported using mosquitoes aged 2–8 days [6, 7, 12, 13, 16, 17, 23–25]. Here we chose to work with 3–5-day-old mosquitoes to ensure that we achieved high survival rates on day 7 for the dissection for oocysts and day 14 for the dissection for sporozoites. Finally, we did not observe any significant difference between the water bath temperatures of 34 °C, 38 °C, 42 °C and 46 °C. While most studies have indicated using a water bath at 37 °C [18, 30], this parameter has not been investigated before. Our results show that mosquitoes were able to feed efficiently regardless of fluctuations in the water bath temperatures. We chose to use a water bath temperature of 37–38 °C, as it closely resembles the human body temperature and will be most conducive for malaria parasite survival.

## Conclusion

By sequentially and systematically varying individual membrane feeding parameters, the blood feeding rate of *Anopheles farauti* s.s. colony mosquitoes was increased significantly to 85%. This highlights the importance of parameter selection and optimization in direct membrane feeding assays. Further work will need to be performed with infected blood to ensure that these parameters result in high infection and survival rates for *An. farauti* s.s. colony mosquitoes using DMFA.

## Supplementary Information

**Additional file 1: Table S1.** Flow chart of the feeding parameters that were tested progressively. The selected parameters are in bold and were used in the subsequent tests

## Data Availability

The raw data pertaining to this manuscript can be obtained from the corresponding author upon request.

## Abbreviations

PNG: Papua New Guinea
DMFA: Direct membrane feeding assay
ON: Overnight
*An. farauti*: *Anopheles farauti*
*An. coluzzii*: *Anopheles coluzzii*
