# Supplementary material for: Optimization of the feeding rate of Anopheles farauti s.s. colony mosquitoes in direct membrane feeding assays
Source: Parasit Vectors. 2021 Jul 7;14:356. doi: 10.1186/s13071-021-04842-y (PMC8261992; doi:10.1186/s13071-021-04842-y)

Supplementary Table S1. Flow chart of the feeding parameters that were tested progressively. The selected parameters are in bold and were used in the subsequent tests.

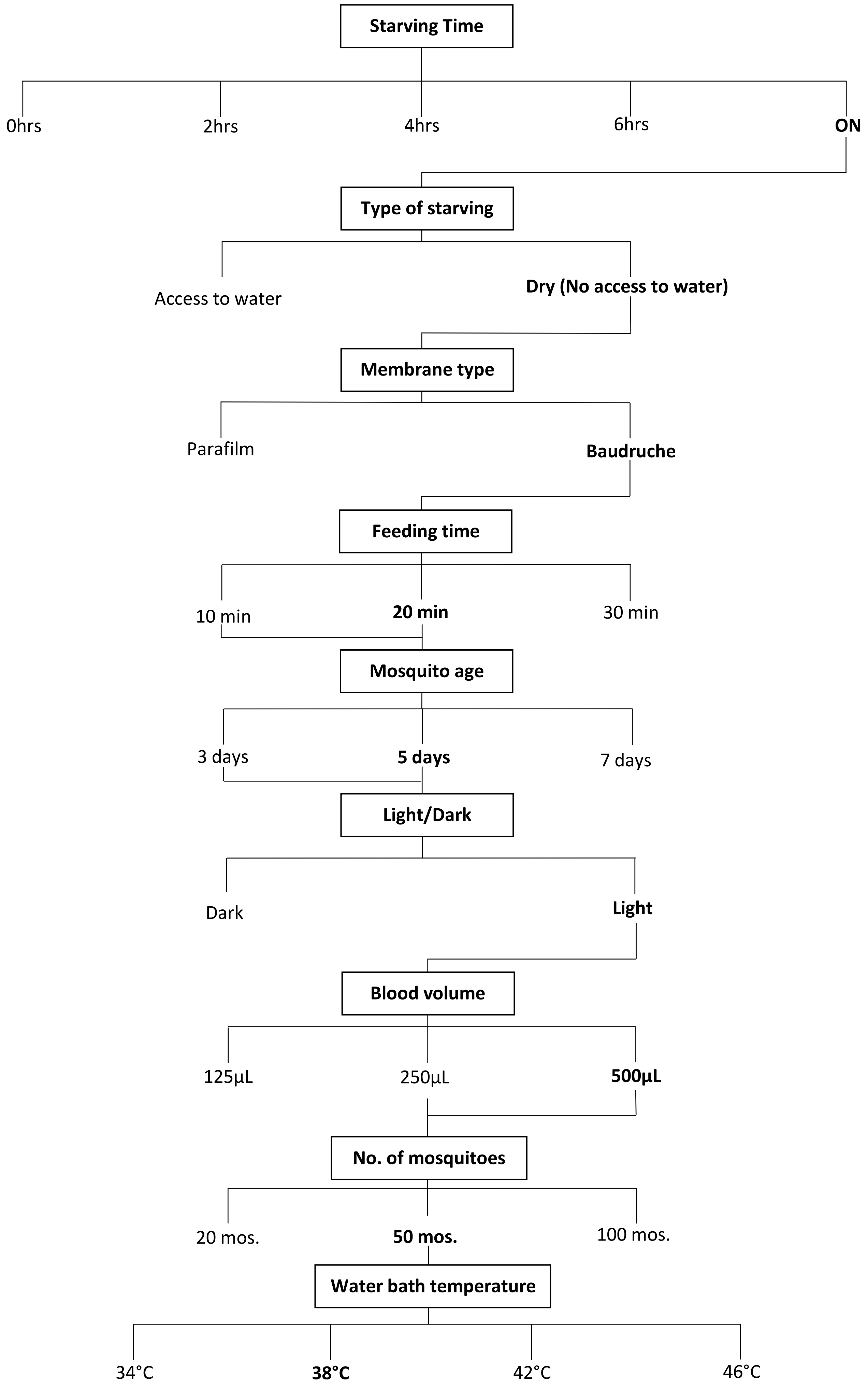

Supplement: Supplementary file 1 — Additional file 1: Table S1. Flow chart of the feeding parameters that were tested progressively. The selected parameters are in bold and were used in the subsequent tests [file 13071_2021_4842_MOESM1_ESM.pdf]
